# Antagonization of the Nogo-Receptor 1 Enhances Dopaminergic Fiber Outgrowth of Transplants in a Rat Model of Parkinson’s Disease

**DOI:** 10.3389/fncel.2017.00151

**Published:** 2017-05-26

**Authors:** Stefanie Seiler, Stefano Di Santo, Lukas Andereggen, Hans R. Widmer

**Affiliations:** ^1^Department of Neurosurgery, Neurocenter and Regenerative Neuroscience Cluster, University Hospital Bern, Switzerland University of BernBern, Switzerland; ^2^Department of Clinical Research, University of BernBern, Switzerland

**Keywords:** Parkinson’s disease, Nogo-receptor 1, cell transplantation, dopaminergic neurons, behavior, rat

## Abstract

Intrastriatal transplantation of fetal human ventral mesencephalic dopaminergic neurons is an experimental therapy for patients suffering from Parkinson’s disease. The success of this approach depends on several host brain parameters including neurotrophic factors and growth inhibitors that guide survival and integration of transplanted neurons. While the potential of neurotrophic factors has been extensively investigated, repression of growth inhibitors has been neglected, despite the significant effects reported in various CNS injury models. Recently, we demonstrated that infusion of neutralizing antibodies against Nogo-A into the lateral ventricles of hemi-parkinsonian rats significantly enhanced graft function. Since the Nogo-receptor 1 also interacts with other neurite growth inhibitors, we investigated whether a direct antagonization of the receptor would result in more robust effects. Therefore, rats with unilateral striatal 6-hydroxydopamine lesions were grafted with ventral mesencephalic tissue in combination with intraventricular infusions of the Nogo-receptor 1 antagonist NEP1-40. Transplanted rats receiving saline infusions served as controls. To test whether NEP1-40 treatment alone affects the remaining dopaminergic striatal fibers, rats with unilateral striatal 6-hydroxydopamine lesions were infused with NEP1-40 or saline without receiving a transplant. Motor behavior was assessed prior to the lesion as well as prior and 1, 3, and 5 weeks after the transplantations. At the end of the experimental period the number of graft-derived dopaminergic fibers growing into the host brain, the number of surviving dopaminergic neurons and graft volume were analyzed. In rats without a transplant, the density of dopaminergic fibers in the striatum was analyzed. We detected that NEP1-40 treatment significantly enhanced graft-derived dopaminergic fiber outgrowth as compared to controls while no effects were detected for graft volume and survival of grafted dopaminergic neurons. Notably, the enhanced dopaminergic fiber outgrowth was not sufficient to improve the functional recovery as compared to controls. Moreover, NEP1-40 infusions in hemi-parkinsonian rats without a transplant did not result in enhanced striatal dopaminergic fiber densities and consequently did not improve behavior. In sum, our findings demonstrate that antagonization of the Nogo-receptor 1 has the capacity to support the engraftment of transplanted mesencephalic tissue in an animal model of Parkinson’s disease.

## Introduction

Parkinson’s disease (PD) is a neurodegenerative disease mainly characterized by progressively degenerating dopaminergic neurons in the substantia nigra pars compacta. The resulting lack of dopaminergic innervation of the striatum and consequent loss of dopamine leads to severe motor symptoms. Cell replacement strategies, i.e., transplantation of human fetal ventral mesencephalic (VM) tissue into the caudate and putamen of patients, that intend to restore the lost dopamine in the striatum, can result in considerable long-term motor improvement up to several years after transplantation (Yasuhara et al., 2006; Hallett et al., 2014; Kefalopoulou et al., 2014; Pantcheva et al., 2015) and are thus a promising experimental approach to treat PD (Bjorklund et al., 2003; Ourednik and Ourednik, 2005; Piquet et al., 2012). For a successful outcome, it is important to assure the survival of grafted dopaminergic cells and their functional integration into the host brain (Emgard et al., 1999; Karlsson et al., 2000; Rath et al., 2013), a process in which the host microenvironment plays a crucial role (Ourednik and Ourednik, 2005; Alsberg et al., 2006; Stefanova et al., 2009; Akbik et al., 2012). The microenvironment consists of the totality of signals induced by local neurotrophic and neurite growth inhibiting factors. The significant improvement in engraftment of VM tissue by administering neurotrophic factors, such as glial cell line-derived neurotrophic factor, brain-derived neurotrophic factor, and neurotrophin 4/5 into the host striatum has been demonstrated years ago (Haque et al., 1996; Rosenblad et al., 1996; Sinclair et al., 1996; Yurek et al., 1996; Yurek, 1998; Espejo et al., 2000; Chaturvedi et al., 2006; Tatard et al., 2007; Thompson et al., 2009). While inhibition of Nogo-A signaling, one of the most potent neurite growth inhibitor in the central nervous system (CNS), has been successfully implemented in various models of CNS injuries (Gonzenbach and Schwab, 2008; Schwab and Strittmatter, 2014), it has so far been largely neglected in studies of cell transplantation approaches for PD. We have recently demonstrated that intraventricular infusion of neutralizing anti-Nogo-A antibodies enhanced survival of grafted dopaminergic neurons and significantly promoted their fiber outgrowth, resulting in an improved functional recovery of the hemi-parkinsonian rats (Seiler et al., 2016). Nogo-A can bind to the Nogo-receptor 1 (NgR1), which then interacts with the leucine rich repeat neuronal protein (LINGO-1) and the low affinity nerve growth factor receptor (p75) and/or the tumor necrosis family member LIMK (TROY) before activating the Rho/ROCK pathway (Schmandke et al., 2014). We and others have shown, that both the neutralization of Nogo-A and the antagonization of the NgR1 complex improve the survival and morphology of dopaminergic neurons. In fact, deletion or antagonization of LINGO-1 enhanced the survival of dopaminergic neurons in an *in vitro* and mouse model of PD (Inoue et al., 2007). Moreover, we could show that antagonization of NgR1 by the peptide NEP1-40 significantly improved the survival of dopaminergic neurons and their morphological complexity in fetal primary VM cultures (Seiler et al., 2013). Based on these observations, we aimed at investigating whether NgR1 antagonization by NEP1-40 improves survival and integration of grafted dopaminergic neurons and functional recovery in a hemi-parkinsonian rat model.

## Materials and Methods

### Animals

Adult female Wistar rats (Janvierlabs, France) were housed at 12 h light dark cycle with food and water *ad libitum*. For the preparation of the transplants, time-pregnant Wistar dams were purchased from Janvierlabs (France). All experiments were carried out in the light phase and in accordance with the guidelines of the Animal Research Ethics Committee of the Canton Berne, Switzerland, and the University of Bern Animal Care and Use Committee, Switzerland.

### Experimental Design

**FIGURE 1:**
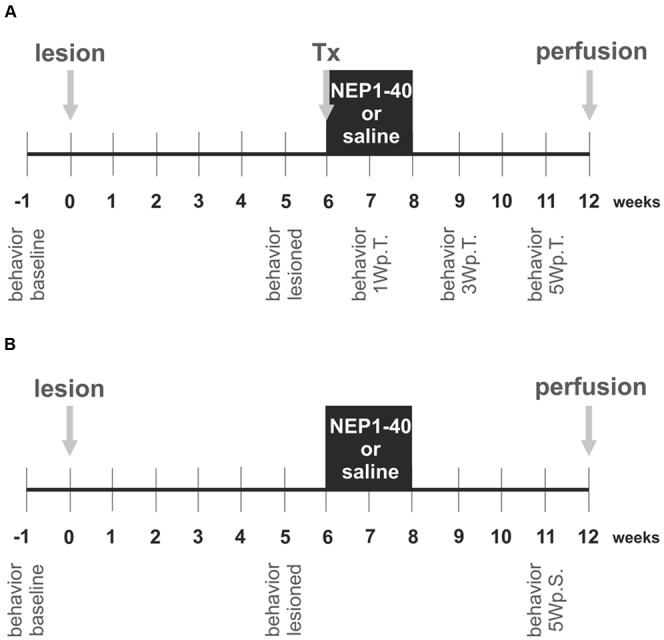
**Experimental design of the study.** The experiments were conducted in two experimental set ups. In the first experiment hemi-parkinsonian rats received a transplant and a mini-osmotic pump **(A)** and in the second experiment hemi-parkinsonian rats received only a mini-osmotic pump and no transplant **(B)**. The neurotoxin 6-hydroxydopamine was injected unilateral into the right striatum of rats (lesion) and 6 weeks later the rats of experimental setup 1 received a transplant of two ventral mesencephalic free-floating roller tube cultures (Tx). Mini-osmotic pumps were implanted into all hemi-parkinsonian rats, releasing either saline or NEP1-40 over a period of 2 weeks into the lateral ventricles (black box). Six weeks after the surgeries rats were transcardially perfused and the brains removed for histological analyses. The cylinder test was assessed in the rats of both the experimental groups, 1 week before the lesion (behavior baseline) and 1 week prior to surgery (behavior lesioned). In the experimental setup 1 behavioral analyzes were performed 1, 3, and 5 weeks post transplantation (behavior 1Wp.T.; behavior 3Wp.T.; behavior 5Wp.T.). In the experimental setup 2 behavior was evaluated 5 weeks after the surgery (behavior 5Wp.S.).

The experimental design is schematically depicted in **Figure 1** and was planned similar to our previous study (Seiler et al., 2016). In the first experimental setup, the hemi-parkinsonian rats received a transplant and a mini-osmotic pump releasing either saline or NEP1-40 into the right lateral ventricles (**Figure 1A**). In the second experimental setup, hemi-parkinsonian rats received only mini-osmotic pumps releasing either saline or NEP1-40 into the right lateral ventricles (**Figure 1B**). To create hemi-parkinsonian rats, 6-hydroxydopamine (6-OHDA) was injected into the right striatum. Six week after the lesions, fetal rat VM tissue was transplanted into the right striatum. In the same surgical procedure, mini-osmotic pumps that continuously infused either saline or NEP1-40 into the right lateral ventricle were implanted (**Figure 1A**). Six weeks after the transplantations rats were perfused and their brains prepared for histological analyses. In these rats asymmetrical forelimb use was assessed by means of the cylinder test before (baseline) and 5 weeks after the lesions (lesioned) and 1, 3, and 5 weeks after the transplantations (**Figure 1A**). In the rats implanted with mini-osmotic pumps only asymmetrical forelimb use was assessed with the cylinder test before the lesion (baseline), 5 weeks after the lesion (lesioned) and 5 weeks after the pump implantation (**Figure 1B**).

### Parkinson’s Disease Rat Model

Female Wistar rats (200–270 g; Janvierlabs, France) were anesthetized with Isoflurane (75% N_2_O, 20% O_2_, 4.5–5%) followed by an intraperitoneal (i.p.) injection of Narketan (75 mg/kg; Vétoquind AG, Ittigen, CH) and Xylazine (5 mg/kg; Vétoquind AG, Ittigen, CH). Buprenorphine (0.5 mg/kg; Reckitt Benckiser AG, Wallisellen) was subcutaneously (s.c.) injected 30 min before the surgical intervention. Under deep anesthesia the rats were placed on a heating pad and fixed in a stereoscopic frame (Stoelting Co.). Through a small burr hole in the skull 4 μl of 6-OHDA (32 mM 6-OHDA; H116 Sigma–Aldrich Chemie GmbH) were injected into the right striatum according to the following coordinates in relation to bregma (Paxinos Watson rat brain atlas): anterior 1.0 mm, lateral 3.0 mm and 5.0 mm ventral to the dura, the incisor bar was set at 0.0 mm. The injection rate was 1 μl/min. The syringe was left in place for additional 4 min before slowly retracting the needle.

### Preparation of Transplants

The tissues for the transplantations were organotypic fetal rat free-floating roller tube cultures prepared from embryonic day 14 (E14) VM as described previously (Andereggen et al., 2009; Seiler et al., 2016). In brief, time-pregnant Wistar rats were anesthetized with Isoflurane (75% N_2_O, 20% O_2_, 4.5–5%) followed by an i.p. injection of Narketan (120 mg/kg) and Xylazine (20 mg/kg) and their fetuses removed by cesarean section. The VM was separated from the brain under a stereoscopic microscope and cut into four equally sized pieced that correspond to two rostral and two caudal portions. Each piece was placed into a gas-permeable conical plastic tube (Falcon) filled with 1 ml of culture medium consisting of 55% DMEM, 32.5% Hank’s balanced salt solution (HBSS; Gibco), 0.3% glucose, 10% fetal calf serum (FCS; Gibco) and 1% 0.01 M HEPES (Merck) as well as antibiotics/antimycotics (No. 061-052 40 D; Gibco). Each tube was positioned in a rotating roller drum that was placed in an incubator at 37°C in a 5% CO_2_ atmosphere as described in detail previously (Spenger et al., 1994). The VM tissue was grown for 7 days and the medium was changed after 2 and 5 days *in vitro*.

### Transplantation and Pump Implantation

Six weeks after the 6-OHDA lesions, rats were anesthetized with Isoflurane (75% N_2_O, 20% O_2_, 4.5–5%) followed by an i.p. injection of Narketan (75 mg/kg; Vétoquinol AG, Ittigen, CH) and Xylazine (5 mg/kg; Vétoquinol AG, Ittigen, CH). Buprenorphine (0.5 mg/kg; Reckitt Benckiser AG, Wallisellen) was s.c. injected 30 min before the surgical intervention. In deep anesthesia the rats were put on a heating pad and mounted on a stereoscopic frame. Half a ventral mesencephalon consisting of one rostral and one caudal part of one embryo was transplanted into the right striatum. This guaranteed approximately equal amounts of dopaminergic neurons per graft. The following coordinates in relation to bregma (Paxinos Watson rat brain atlas) were used: anterior 1.0 mm, lateral 2.7 mm and 4.5 mm ventral to the dura, the incisor bar was set at 0.0 mm. Next, mini-osmotic pumps (2 ml^2^, Alzet osmotic pumps, DURECT Corporation ALZET Osmotic Pumps) pre-filled with saline or NEP1-40 (75 μg/kg/day) (GrandPre et al., 2002) were implanted under the skin of all the rats and the cannulas were subcutaneously connected to the skull and placed into the right ventricles (Alzet brain infusion kit2) according to the following coordinates in relation to bregma (Paxinos Watson rat brain atlas): posterior 0.8 mm, lateral 1.6 mm and 3.5 mm ventral to the dura, the incisor bar was set at 0.0 mm. The substances were administered by the mini-osmotic pumps continuously over the subsequent 2 weeks with a flow rat of 5 μg/h. The animals were randomly assigned to the two treatment groups (saline *n* = 6; NEP1-40 *n* = 7). Rats from the non-grafted groups (experimental setup 2) experienced the same mini-osmotic pump surgeries (saline *n* = 7; NEP1-40, *n* = 7). Animals were let to recover for 1 week after the surgery.

### Behavior Test Series

The cylinder test is a reliable measure to assess the asymmetry in forelimb use as observed after a unilateral lesion of the dopaminergic nigro-striatal pathway (Brooks and Dunnett, 2009; Schaar et al., 2010) and was evaluated as previously described (Seiler et al., 2016). In brief the rats were placed in a transparent cylinder (diameter 30 cm and height 41 cm) with mirrors placed around it to allow a 360 degree view on the cylinder walls. The 10 min video recordings of the rat’s behavior were analyzed by a researched blinded to the treatment groups by counting the number of wall touches with the left, the right and both paws together. To discriminate between a meaningful physiological movement and an accidental touch, only wall contacts by which the rat supported its body weight on the forelimb with extended digits were counted. One animal in the control group had to be excluded from the analysis as it did not touch the wall at all after the lesion. The percentage of left wall touches are calculated according to the formula: [(left + ½ of both paw touches)/(left + right + both paw touches)] ^∗^ 100 as previously described (Boix et al., 2015; Seiler et al., 2016).

### Perfusions

Six weeks after the transplantation, rats were anesthetized with Isoflurane (75% N_2_O, 20% O_2_, 4.5–5%) followed by an i.p. injection of Narketan (75 mg/kg) and Xylazine (5 mg/kg). Fentanyl (0.005 mg/kg, Janssen-AG, Zug, CH) was i.p. injected just prior to opening the thorax and the rats were perfused with 200 ml ice cold 0.1 M phosphate buffer saline (PBS, pH 7.4) containing heparin (1000 I. E./100 ml, NOVO Nordisk) followed by 250 ml 4% paraformaldehyde in 0.1 M PBS. The brains were removed from the skull and placed in 4% paraformaldehyde overnight and thereafter cryoprotected in 10% sucrose-PBS solution.

### Immunohistochemistry

Immunohistochemistry was performed as described previously (Seiler et al., 2016). The brains were cut at 30 μm on a cryostat (Leica CM 1900) and mounted on Superfrost slides (Thermo Scientific). Sections were heated in citrate buffer for 30 min and blocked with 10% horse serum in 0.1% Triton-PBS. Primary and secondary antibodies were incubated in a 0.1% Triton-PBS solution containing 2.5% horse serum. Slides were incubated with the mouse monoclonal anti-tyrosine hydroxylase (TH; 1:1000, Millipore) overnight. After PBS washes, sections were incubated for 2 h with the secondary biotinylated anti-mouse IgG antibody (1:200, Vector Laboratories) and the endogenous peroxidase blocked with a solution of 10% methanol and 3% hydrogen peroxide in PBS. Thereafter, the slides were incubated with an avidin-biotin-complex (7 μl/ml; Vectastain ABC-Peroxidase KIT, Vector Labs) for 1 h and specifically bound antibodies were visualized with a metal-enhanced 3,3′-diaminobenzidine substrate kit (Pierce, 34002, Life Technologies). The sections were dehydrated in alcohol, cleared in xylene and mounted in Eukitt (O. Kindler GmbH, Freiburg, Germany).

### Histological Analyses

All analyses were done by a researcher blinded to the treatment groups.

#### Estimation of TH Positive Cells in the SNc

The estimation of the extent of the lesion was done as described previously (Tronci et al., 2012; Seiler et al., 2016). In brief, brain sections of each rat were chosen that match the following coordinates in relation to bregma (Paxinos Watson rat brain atlas): posterior 4.8 mm (three sections analyzed, range 4.7–4.9 mm), 5.3 mm (three sections analyzed, range 5.2–5.4 mm), and 5.8 mm (three sections analyzed, range 5.7–5.9 mm). A light microscope equipped with a motorized stage and a video camera connected to a PC was used for counting. After delineation of the SNc with a 1× objective, the CAST system (Visiopharm) generated an unbiased counting frame with the 40× objective within the delineated SNc area. Only TH positive cells with a clearly stained cell body that were within the counting frame were counted. Data are expressed as percentage of TH positive cells on the lesioned side as compared to the number of TH positive neurons on the unlesioned side.

#### Estimation of the Graft Volume

Every third section containing a graft was selected to determine the size of the graft as previously reported (Andereggen et al., 2009; Seiler et al., 2016). In brief, an Olympus microscope (Olympus DP72) equipped a digital camera and connected to a PC with a calibrated neuron tracing software (CellSens Dimension, Olympus) was used to trace the graft boundaries. Thereafter, an automated computation integrated the areas to yield the graft volume. Graft volume in control animals was 0.21 ± 0.03 mm^3^ (mean ± SEM; *n* = 6).

#### Estimation of the TH Positive Fibers Growing into the Host Brain

Every third section containing a graft was selected to determine the number of TH positive fibers growing 100 μm into the host brain as previously described (Seiler et al., 2016). In brief, an Olympus microscope (Olympus DP72) equipped a digital camera and connected to a PC with a calibrated neuron tracing software (CellSens Dimension, Olympus) was used to draw a virtual line 100 μm from the graft boundary with a length of 300 μm. TH positive fibers originating in the graft were followed up to this virtual line and counted if they crossed this line. The counts were done at four sites, i.e., medial, lateral, dorsal, and ventral from the boarder of the graft using a 10× objective and verified with a 40× objective. Number of TH positive fibers in control animals were 5.5 ± 1.1, 7.7 ± 0.4, 4.6 ± 0.9, and 5.8 ± 1.1 crossing the virtual line at the medial, lateral, dorsal and ventral sites of the graft, respectively (mean ± SEM; *n* = 6).

#### Estimation of the TH Positive Cells in the Graft

Every third section containing a graft was selected to determine the number of TH positive cells in the graft using the optical fractionators design as described by West et al. (1991), Kelsen et al. (2010). An Olympus light microscope (Olympus DP72) equipped with a motorized stage and a digital camera connected to a PC was used for stereological counting. After delineation of the graft with a 4× objective, the TH positive cells with a clearly stained cell body were counted with a 40× objective. The estimated cell number was calculated by the following equation *N*: = (1/ssf)^∗^(1/asf)^∗^(1/hsf)^∗^the sum of the counts as described by Kelsen et al. (2010). The section sample fraction (ssf) was 0.3, because every third section was used for analysis. The area sampling fraction (asf) was set to 1 as 100% of the graft was analyzed. The height of the sampling fraction (hsf) was 1 as the whole section thickness of 30 μm was used. Grafts of controls contained 547 ± 71 TH positive neurons (mean ± SEM; *n* = 6).

#### Estimation of Remaining TH Positive Fibers in the Striatum

One section of each rat at the following coordinate in relation to bregma (Paxinos Watson rat brain atlas): anterior 1.5 mm was selected to determine the remaining TH positive fiber density in the striatum of rats that did not receive a transplant (second experimental setup), as described previously (Seiler et al., 2016). In brief, the striatum of the lesioned and unlesioned side was photographed by a microscope (Olympus DP72) that was connected to a digital camera (Olympus). The images were converted to 8 bit black and white pictures and inverted using the Fiji software. The mean gray intensity of the dorsal striatum of the lesioned and unlesioned side was measured in a defined area (100,000 μm^2^). To account for non-specific background staining, the mean gray value in a defined area (30,000 mm^2^) in the corpus callosum was measured. The mean gray intensity of the corpus callosum was subtracted from the respective striatal mean gray intensity values and then the values of the lesioned striatum were expressed as percentage of the unlesioned side.

### Statistical Analysis

For statistical analysis a commercially available software package was used (GraphPad Prism 7). To compare group means of several groups repeated two-way ANOVA was used, followed by Tukey’s or Bonferroni’s multiple comparison test where appropriate. Statistical significance of two groups only, was assessed by two-tailed unpaired *t*-test and the statistical significance levels are expressed as tα/υ, where α indicates the student’s *t*-values and υ the relative degree of freedom. Statistical significance was set at *p* < 0.05. Data are presented as mean ± SEM.

## Results

### Estimated Extent of the Lesion

The analysis of TH immunoreactive neurons in the SNc disclosed a lesion extent of 68% ± 3.4% in the first experimental setup and 36.4% ± 6.0% in the second. No significant difference between the three levels analyzed or between the saline and NEP1-40 treatment groups was detected.

### Behavioral Analyses

#### Cylinder Test Experimental Set Up 1

**FIGURE 2:**
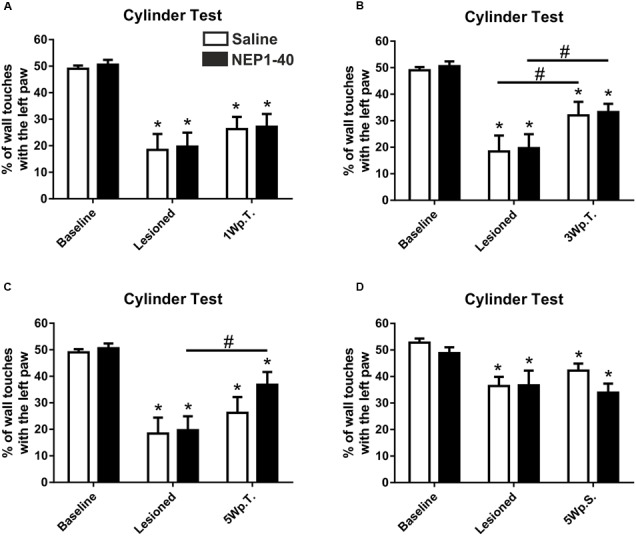
**Asymmetrical forelimb use of saline or NEP1-40 treated rats assessed with the cylinder test.** The contralateral forelimb use (left paw use) is shown before (baseline) and after the 6-hydroxydopamine lesions as well as 1 (1Wp.T.; **A**), 3 (3Wp.T.; **B**) and 5 weeks post transplantation (5Wp.T.; **C**) in the experimental setup 1. The lesions resulted in a significantly reduced number of wall touches with the left paw as compared to baseline levels **(A–C)**. Animals exposed to NEP1-40 treatment gradually improved the asymmetrical forelimb use over the 5 weeks period **(B,C)**, however, did not reach baseline level **(A–C)**. While an overall similar outcome was observed for the saline treated group at 5Wp.T. these animals did not perform significantly better than at the post-lesion time point **(C)**. In the experimental setup 2, no behavioral recovery was found 5 weeks after pump implantation (5Wp.S) in both groups **(D)**. Data are given as mean + SEM and expressed as percentage of left paw use. ^∗^*p* < 0.05 vs. corresponding baselines, ^#^*p* < 0.05 vs. corresponding lesion levels.

The asymmetrical forelimb use after unilateral 6-OHDA lesion was assessed in a cylinder. As expected, the lesioned rats scored significantly less often with the left paw (contralateral to the lesion) compared to baseline (49.0 ± 1.2 vs. 18.4 ± 6.0, % of left paw use for baseline and lesioned in the saline group, *t*_4.9/4_ ≤ 0.01 and 50.6 ± 1.8 vs. 19.7 ± 5.2, % of left paw use for baseline and lesioned in the NEP1-40 group, *t*_6.0/6_ ≤ 0.001) (**Figure 2A**). No significant difference could be found between NEP1-40 and saline treated groups (data not shown). One week after the transplantation no significant behavioral improvement was observed as compared to the post-lesion time point [26.3 ± 4.6 vs. 18.4 ± 6.0 and 27.2 ± 4.8 vs. 19.7 ± 5.2; % of left paw use for saline and NEP1-40 vs. lesioned, respectively; Time point, *F*(2,20) = 41.29, *p* < 0.0001; *post hoc*, lesioned vs. saline *p* = 0.33 and lesioned vs. NEP1-40 *p* = 0.25] (**Figure 2A**). In contrast, 3 weeks after the transplantation the rats significantly increased the use of the left paw in both treatment groups as compared to the post-lesion time point [32.0 ± 5.1 vs. 18.4 ± 6.0 and 33.3 ± 3.1 vs. 19.7 ± 5.2; % of left paw use for saline and NEP1-40 vs. lesioned, respectively; Time point, *F*(2,20) = 38.72, *p* < 0.0001; *post hoc*, lesioned vs. saline *p* = 0.05 and lesioned vs. NEP1-40 *p* = 0.02] (**Figure 2B**). On the other hand, only the NEP1-40 treated rats showed a significant constant improvement in left paw use over the 5 weeks after transplantation as compared to lesioned (36.8 ± 4.8 vs. 19.7 ± 5.2; % of left paw use for NEP1-40 and lesioned, respectively; Time point, *F*(2,20) = 28.19, *p* < 0.0001; *post hoc*, lesioned vs. NEP1-40 *p* = 0.01] (**Figure 2C**). In contrast, saline treated rats did not perform significantly better 5 weeks after the transplantation than at the post-lesion time point [26.2 ± 5.9 vs. 18.4 ± 6.0; % of left paw use for saline and lesioned, respectively; Time point, *F*(2,20) = 28.19, *p* < 0.0001; *post hoc*, lesioned vs. saline *p* = 0.44] (**Figure 2C**). Hence, even though both treatment groups showed a tendency for recovery, at all observed time points all rats performed significantly worse compared to baseline (**Figures 2A–C**). Moreover, the NEP1-40 treated rats never displayed significantly better results in the behavioral test than the saline treated rats (1Wp.T. 27.2 ± 4.8 vs. 26.3 ± 4.6; 3Wp.T. 33.3 ± 3.1 vs. 32.0 ± 5.1 and 5Wp.T. 36.8 ± 4.8 vs. 26.2 ± 5.9; % of left paw use for NEP1-40 and saline treated rats; Treatment, *F*(1,10) = 0.07; *p* > 0.05; *post hoc*, 1Wp.T. saline vs. NEP1-40 *p* = 1.0; Treatment, *F*(1,10) = 0.12; *p* > 0.05; *post hoc*, 3Wp.T. saline vs. NEP1-40 *p* = 1.0; Treatment, *F*(1,10) = 1.01; *p* > 0.05; *post hoc*, 5Wp.T. saline vs. NEP1-40 *p* = 0.34] (**Figures 2A–C**).

#### Cylinder Test Experimental Set Up 2

The lesioned rats scored significantly less often with the left paw (contralateral to the lesion) compared to baseline (52.8 ± 1.6 vs. 36.4 ± 3.5, % of left paw use for baseline and lesioned in the saline group, *t*_6.6/6_ ≤ 0.01 and 48.8 ± 2.2 vs. 36.7 ± 5.5, % of left paw use for baseline and lesioned in the NEP1-40 group, *t*_3.2/6_ ≤ 0.01) (**Figure 2D**). No significant difference could be found between NEP1-40 and saline treated groups (data not shown). Five weeks after the transplantation no significant behavioral improvement was observed as compared to the post-lesion time point [42.2 ± 2.7 vs. 36.4 ± 3.5 and 33.9 ± 3.4 vs. 36.7 ± 5.5; % of left paw use for saline and NEP1-40 vs. lesioned, respectively; Time point, *F*(2,24) = 13.26, *p* < 0.0001; *post hoc*, lesioned vs. saline *p* = 0.38 and lesioned vs. NEP1-40 *p* = 0.80] (**Figure 2D**). Moreover, the rats touched the wall with the left paw still significantly less often (**Figure 2D**).

### Histological Analyses

**FIGURE 3:**
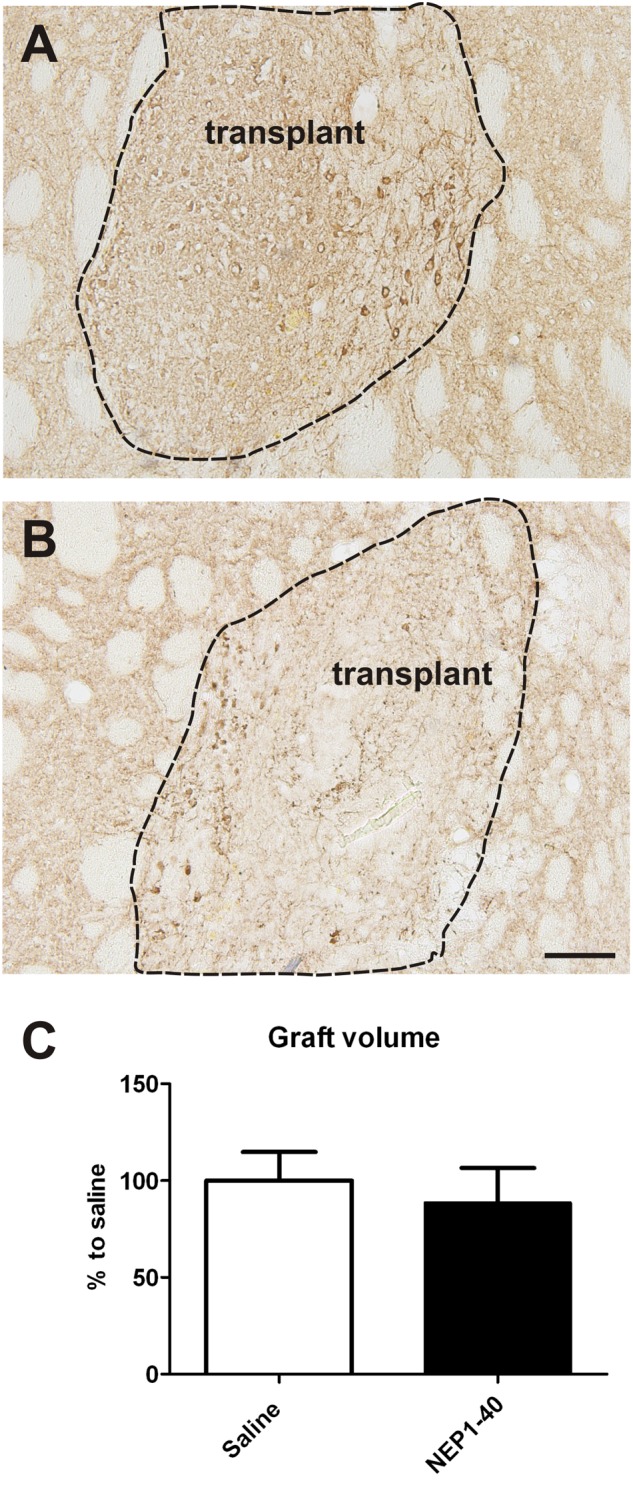
**Effects of intra-ventricular infusion of saline or NEP1-40 on graft volumes.** Representative photomicrographs of intrastriatal grafts treated with either saline **(A)** or NEP1-40 **(B)** stained for tyrosine hydroxylase. The graft boarder is highlighted with a dashed black line. Scale bar: 100 μm. No difference in graft volume was detected between the two treatment groups, as shown in the bar graph **(C)**. Data are given as mean + SEM and are presented as percentage of saline.

**FIGURE 4:**
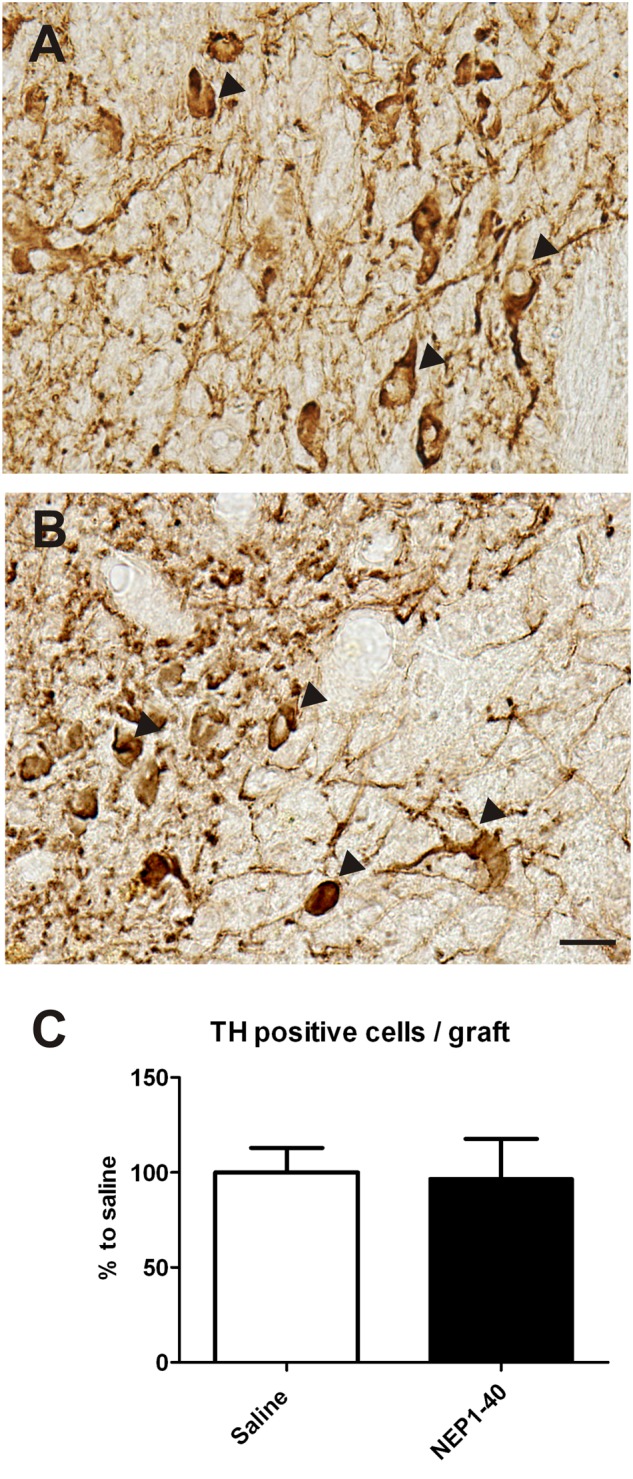
**Effects of intra-ventricular infusion of saline or NEP1-40 on tyrosine hydroxylase (TH) positive cell numbers in the grafts.** Representative photomicrographs of TH positive neurons (arrowheads) in the grafts of saline **(A)** and NEP1-40 treated **(B)** rats. Scale bar: 20 μm. No difference between the two groups could be observed, as depicted in the bar graph **(C)**. Data are given as mean + SEM and are presented as percentage of saline.

**FIGURE 5:**
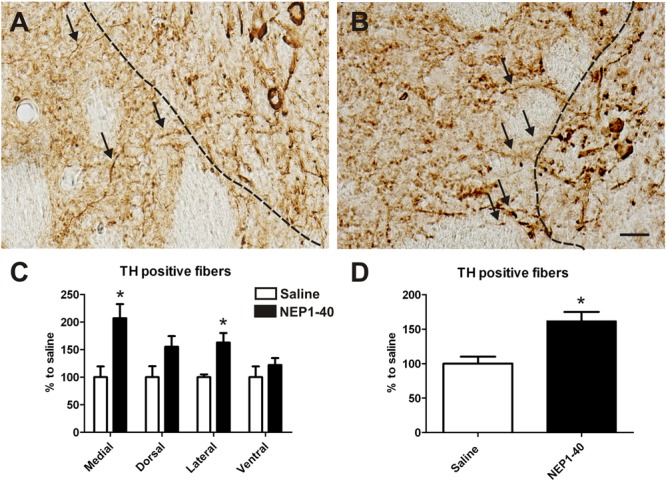
**Effects of intra-ventricular infusion of saline or NEP1-40 on tyrosine hydroxylase (TH) positive fiber outgrowth.** Representative photomicrographs of TH positive fibers from the graft growing into the host tissue (arrows) from saline **(A)** and NEP1-40 treated **(B)** animals. The black dashed line indicates the graft boarder. Note the slight but significant increase in TH positive fibers growing 100 μm medially into the host brain resulting in a significant overall larger TH positive fiber outgrowth into the host brain, as indicated in the bar graphs **(C,D)**. Scale bar: 20 μm. Data are given as mean + SEM and are presented as percentage of corresponding saline. ^∗^*p* < 0.05.

**FIGURE 6:**
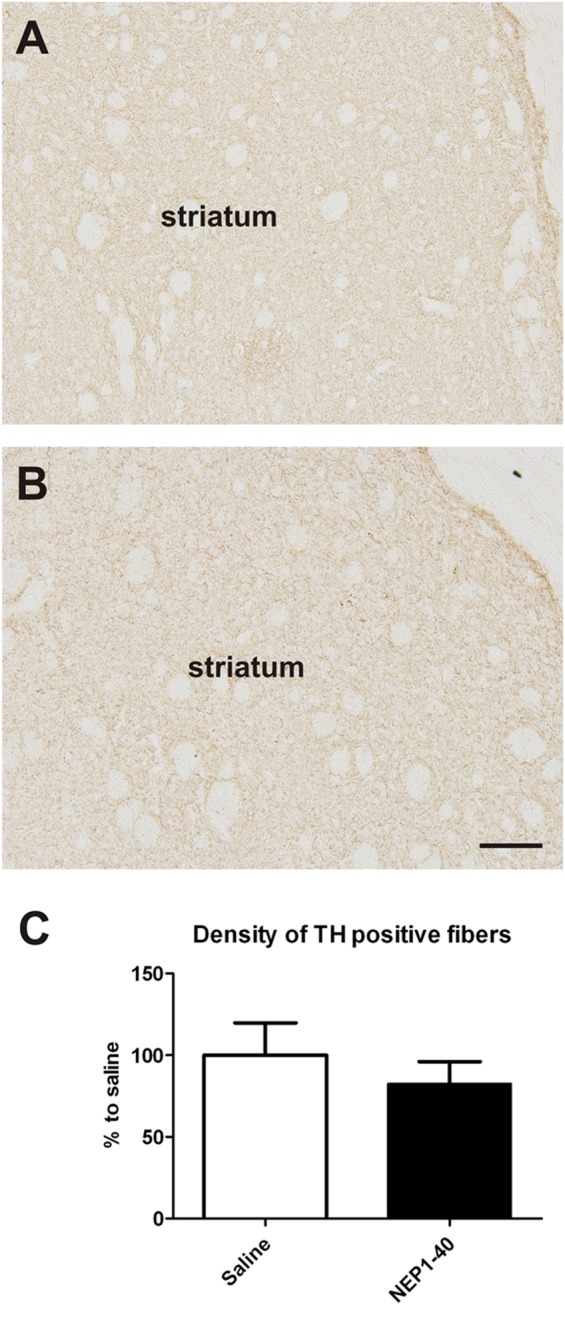
**Effects of intra-ventricular infusion of saline or NEP1-40 on striatal tyrosine hydroxylase (TH) positive fiber density.** Representative photomicrographs of TH positive fiber densities in the striatum from saline **(A)** and NEP1-40 **(B)** treated animals. No difference in striatal TH positive fiber density was detected between the two groups **(C)**. Scale bar: 100 μm. Data are given as mean + SEM and are presented as percentage of saline.

At the histological level the graft volume did not differ in both treatment groups (88.6% ± 18.0% vs. 100% ± 14.7%; for NEP1-40 and saline treatment, respectively; *t*_0.48/11_ = 0.64) (**Figure 3**). Likewise, the content of surviving TH positive cells per graft was similar in the two experimental groups (96.9% ± 20.9% vs. 100% ± 12.9%; for NEP1-40 and saline treatment; *t*_0.12/11_ = 0.90) (**Figure 4**). However, NEP1-40 treatment significantly promoted the fiber outgrowth of transplanted TH positive neurons into the host brain as compared to saline treatment (by 1.6-fold; *t*_3.6/11_ = 0.004). A more detailed analysis revealed that significantly more TH positive fibers in the NEP1-40 group grew medially and laterally into the host brain as compared to the saline group [*F*(1,11) = 13.24; *p* < 0.05; *post hoc, p* < 0.001 and *p* < 0.01, respectively], whereas dorsal and ventral sites did not show a significant difference between the groups (**Figure 5**). In experimental setup 2 in which rats were receiving mini osmotic pumps only, no difference in striatal TH positive fiber densities was detected between saline and NEP1-40 treated animals (*t*_0.5/12_ = 0.621) (**Figure 6**).

## Discussion

This study shows that NgR1 antagonization in combination with tissue grafts significantly promoted TH positive fiber outgrowth into the host brain of hemi-parkinsonian rats. Moreover, over the post transplantation period the NEP1-40 treated group displayed a tendency for behavioral improvement, however, this did not reach statistical significance. These observations hint to the idea that a certain level of TH positive fibers growing into the host brain is needed to result in behavioral recovery. In line with this notion, our correlation analyzes for TH positive neurons in the graft and TH positive fiber outgrowth with asymmetrical forelimb use showed no association (data not shown). Our results underscore the critical role of the number of surviving TH positive neurons in the grafts for the improvement of the behavioral deficits within the frame of the experimental conditions described here (Brundin et al., 1988; Meyer et al., 1998). The only partial functional recovery observed in the saline treated control rats grafted with half a VM was expected and purposely chosen to allow monitoring of the efficacy of the NEP1-40 treatment (Meyer et al., 1998; Seiler et al., 2016). The lack of effect of NEP1-40 treatment on the TH cell number in the transplants and on the graft volume is, however, in contrast to our previous work, demonstrating an increase in volume and number of TH positive cells in VM free-floating roller tube cultures (Seiler et al., 2013). This difference might depend on the actual concentration of NEP1-40 reaching the VM tissue. While in the culture system the NEP1-40 concentration is defined and stable, NEP1-40 infused into the lateral ventricle is likely diluted in the CSF given the continuous turnover of the CSF (3.4 l/min (Redzic et al., 2005)). NEP1-40 does diffuse from the CSF into the brain parenchyma as several studies have shown (Fang et al., 2010; Wang et al., 2012). The observation that the effects of NEP1-40 treatment on TH positive fiber outgrowth was most prominent toward the ventricle further support these findings. Nevertheless, the effects of NEP1-40 on VM tissue grafts in our study might have been underestimated, even though we have used a concentration as previously described (Wang et al., 2012), and further studies using higher concentrations of NEP1-40 are needed. Given that in our experimental model the striatum is not completely denervated by 6-OHDA and NEP1-40 penetrates into the parenchyma, we next reasoned whether NEP1-40 might target the spared TH fibers. In experimental setup 2 we tested for that hypothesis. In our measurements of TH positive fiber densities, however, we found no evidence that NEP1-40 exerts effect on TH fiber densities in absence of a graft. Importantly to note, we cannot exclude that NEP1-40 treatment exerts other effects on the brain, such as for example on microglia (Fang et al., 2015).

These results complement our previous findings gathered using the same experimental setup investigating the potential of inhibition of Nogo-A/NgR1 signaling by means of ventricular infusion of neutralizing Nogo-A antibodies (Seiler et al., 2016). So, we reported that anti-Nogo-A antibody treated rats gradually improved their performance in the cylinder test, reaching almost baseline levels 5 weeks after the transplantation (Seiler et al., 2016). In this respect it is important to note that NEP1-40 treatment seems to have a substantially lower capacity to support graft function. We hypothesize that the different signaling partners and mechanisms activated by Nogo-neutralization and by its receptor antagonization might account, at least in part, for the dissimilar effects observed in the two distinct studies. In fact, the neutralizing anti-Nogo-A antibodies used in the previous work targeted the Δ-20 domain of Nogo-A, which is known to bind to the sphingosine-1 phosphate receptor 2 (S1PR2) but not to the NgR1 (Akbik et al., 2012; Kurowska et al., 2014; Saha et al., 2014). Hence, functional recovery and increased TH positive fiber outgrowth after anti-Nogo-A antibody infusion in our previous study was observed even though the Nogo-A-NgR1 pathway was still active. Given that the NgR1 does not only bind Nogo-A through its Nogo-66 domain, but also other growth inhibitors such as myelin associated glycoprotein, the oligodendrocyte myelin glycoprotein and the chondroitin sulfate proteoglycans (Akbik et al., 2012; Schwab and Strittmatter, 2014), it was tempting to speculate that antagonization of the NgR1 might be more potent in enhancing graft function and TH positive fiber outgrowth than inhibiting specifically Nogo-A. The results of the present study, however, did not support this notion even though the above mentioned issues need to be addressed in more depth to draw a final conclusion.

## Conclusion

In sum, the present study demonstrates that NEP1-40 treatment of hemi-parkinsonian rats promoted TH positive fiber outgrowth into the host brain which, however, was not sufficient to induce functional recovery to baseline levels. In line with the latter observation NEP1-40 treatment did not enhance graft volume or survival of transplanted dopaminergic neurons. Alternative strategies to inhibit Nogo-A signaling as for example through neutralizing anti-Nogo-A antibodies possibly in combination with application of neurotrophic factors should be considered to enhance engraftment and function of the transplant.

## Author Contributions

Author’s contribution to the study and manuscript preparation includes the following. Conception and design of the work was done by SS, SD, and HW. Acquisition of data was performed by SS, SD, LA, and HW. Analysis and interpretation of data was carried out by SS, SD, LA, and HW. The draft of the article was performed by SS and critically revised by SD and HW. All authors read and approved the final manuscript.

## Conflict of Interest Statement

The authors declare that the research was conducted in the absence of any commercial or financial relationships that could be construed as a potential conflict of interest.
